# DEVELOPING THE INTERGENERATIONAL TECHNOLOGY ENGAGEMENT CHECKLIST FOR HIGHER EDUCATION (IG TECH)

**DOI:** 10.1093/geroni/igae098.0185

**Published:** 2024-12-31

**Authors:** Jill Juris, Skye Leedahl, Athena Sexton, Josie Santilli, Meaghan Colvin

**Affiliations:** Appalachian State University, Boone, North Carolina, United States; University of Rhode Island, Kingston, Rhode Island, United States; Appalachian State University, Boone, North Carolina, United States; University of Rhode Island, Kingston, Rhode Island, United States; University of Rhode Island, Kingston, Rhode Island, United States

## Abstract

Intergenerational programs, as part of service learning initiatives in higher education, have shown to greatly benefit students in gaining professional experience that connects with classroom learning. In recent years, intergenerational technology programs in the United States and Canada have worked to enhance digital inclusion for older adults and provide service learning opportunities for students. Funded by the RRF Foundation for Aging, our multi-university research team set out to examine the implementation of programs that utilized the Cyber-Seniors reverse mentoring model within higher education. This presentation will describe the creation of a checklist to aid in the development of intergenerational technology programs within higher education, with the goal of building sustainable programs. The checklist was developed following a systematic review of the literature and interviews with 12 stakeholders. The systematic review included the analysis of 17 sources that addressed key elements of intergenerational programs such as planning, technology, accessibility, inclusivity, recruitment, marketing, safety, implementation, staffing, and training. Interviews were conducted with a purposive sample of ten higher education representatives and two Cyber-Seniors staff members, all with extensive knowledge of barriers and facilitators to program implementation. This resulted in the development of the InterGenerational Technology Engagement Checklist for Higher education (IG TECH). The IG TECH includes a series of questions to help faculty and/or staff make decisions about key elements of the program, including program planning, student recruitment, older adult recruitment and onboarding, and implementation/monitoring. Future research will evaluate the checklist through a Delphi Panel.

